# Cryptorchidism in Equid: A Retrospective Study on Diagnostic Approach, Anaesthesia Management, Surgical Treatment and Outcomes

**DOI:** 10.3390/ani15192923

**Published:** 2025-10-09

**Authors:** Irene Nocera, Rebecca Moroni, Diana Fanelli, Alessandra Rota, Chiara Di Franco, Camilla Ungari, Caterina Puccinelli, Marco Gandini, Gessica Giusto, Iacopo Vannozzi

**Affiliations:** 1 Institute of Health Sciences, School of Advanced Study Sant’Anna, 56127 Pisa, Italy; 2 Department of Veterinary Sciences, University of Pisa, 56124 Pisa, Italy; rebe.moroni@gmail.com (R.M.); diana.fanelli@unipi.it (D.F.); alessandra.rota@unipi.it (A.R.); c.ungari@studenti.unipi.it (C.U.); caterina.puccinelli@unipi.it (C.P.); iacopo.vannozzi@unipi.it (I.V.); 3 Institute of Clinical Physiology, Italian National Research Council, 56124 Pisa, Italy; chiaradifranco@cnr.it; 4 Equine Health Center, 10046 Torino, Italy; marco.gandini@unito.it (M.G.); gessica.giusto@unito.it (G.G.)

**Keywords:** equid, cryptorchidism, flank laparoscopy, ultrasound

## Abstract

**Simple Summary:**

Cryptorchidism is a sexual development commonly encountered in equid males. This study retrospectively analyses cases of cryptorchidism in equid referred to specialised centres, including horses, a donkey and a female intersex horse. This study provides specific information on breed, diagnostic approach, surgical treatment, anaesthesia plan, surgical complications and follow-up. The findings add information on this condition, widely spread among equid, and the possible surgical approaches.

**Abstract:**

Over the years, various diagnostic and surgical techniques have been developed to recognise and treat cryptorchidism in equid. This study retrospectively analysed cases of cryptorchidism referred to two centres between 2012 and 2025. Clinical data collected included species, breed, age, gender, location of retained testis, diagnostic approach, anaesthesia plan and surgical treatment, perioperative medical treatment, intra-operative complications and outcomes. A total of 37 clinical cases were included, 1/37 was a donkey and 1/37 was an intersex female. Most of the horses were three years old and belonged to western riding horse breeds. Ultrasound examination correctly identified the location of the retained testis in 87% of cases. The anaesthesia plan includes a local anaesthesia block, mainly a local injection for the infiltration of the retained testis. The laparoscopic standing flank technique was the main approach, performed in 82% of cases. Complications were encountered in 9% of cases, and the median discharge time was 2 days. In our study, Quarter Horse-type horses were overrepresented, and three horses were sons of the same Quarter Horse stallion, suggesting a genetic component in aetiology. The use of ultrasound might confirm cryptorchidism in both abdominal and inguinal locations. Standing flank laparoscopy was the most frequently chosen procedure, over recumbent laparoscopy and inguinal open orchiectomy. The outcome reported a low rate of intra-operative complications.

## 1. Introduction

Cryptorchidism in equids is associated with hypofertility and predisposition to the development of testicular neoplasms, which may negatively affect both reproductive and athletic performance [1].

The testis can be retained either inguinally or abdominally. It is reported in 5–8% of male foals, where one or both testes failed to descend [2]. The left testis is most commonly involved (55%), and it is mainly retained in the abdominal location (75%, 41% of all cryptorchid testes), compared to the right abdominal retained testes (24%) [1]. Bilateral cryptorchidism is less commonly reported (9–12%); however, the abdominal location is prevalent [1,3].

Etiopathogenesis remains poorly understood and may involve both genetic and environmental factors [3]. Cryptorchidism is a common congenital abnormality and may be heritable, although clear evidence for heredity is lacking. Reported genetic associations include autosomal dominant, autosomal recessive or sex-linked inheritance patterns [1,3]. Anatomical abnormalities of the gubernaculum, vaginal process or inguinal canal are also involved in cryptorchidism aetiology [4]. Breed predispositions have been noted, with higher prevalence reported in Percheron, American Saddle Horse and Quarter Horse [1,3].

Over the years, several diagnostic and surgical techniques have been developed to treat cryptorchidism in equids [1,5]. The diagnosis of cryptorchidism is usually based on the results of physical examination, including palpation per rectum, ultrasound and, in selected cases, hormonal assays (testosterone/oestrogen), which could confirm the presence of testicular tissue.

Refs. [1,5] revealed that the most commonly performed technique is transrectal examination, which predicts the correct location of the testis with a high accuracy (87.9%). The inguinal and transabdominal ultrasonographic approaches have reported a sensitivity of 97.5% when combined. The association of transrectal and inguinal ultrasonography can reach a high sensitivity, with a 100% correlation with surgical diagnosis [1,5].

In cases where the standard diagnostic approach failed to locate the retained testis, direct visualisation with a surgical laparoscopy approach has also been reported [1,5]. Laparoscopic cryptorchidectomy is preferred, whenever possible, as it is a minimally invasive surgical approach. This surgical approach is also considered the technique of choice in several different species, such as dogs and cats [6,7], ruminants [8] and llamas [9]. It is reported a favourable post-operative outcome, and a progressive subsidence of stallion-like behaviour. This study aimed to conduct a retrospective analysis of equine cases referred to two equine referral centres for cryptorchidism between 2012 and 2025. It aimed to confirm previous findings and provide further information on a distinct equid population referred to the specialised equid centres to enhance and strengthen the generalizability of both current and erlier results. The present study provides special emphasis on clinical procedures for the determination of the diagnostic technique for the localisation of the retained testes, surgical procedure and local anaesthesia technique.

## 2. Materials and Methods

### 2.1. Cases

The clinical records of equid referred, between 2012 and 2025, for cryptorchidism at two different equine referral centres (Veterinary Teaching Hospital, University of Pisa, Italy and Equine Health Centre, Torino, Italy) were reviewed. The owner’s written consent was obtained. The equid referred to the centres diagnosed with uni- or bilateral cryptorchidism were enrolled.

The following data were collected from clinical records: history signalment (species, breed, age, gender), location of the retained testis and diagnostic approach, anaesthesia plan and type of surgical treatment, pre- and post-operative medical treatment, intra-operative complications and outcomes.

### 2.2. Diagnostics

All equids underwent a thorough physical examination. During the clinical examination, the subjects were restrained in a stock, and if necessary, the subjects were sedated with an intravenous (IV) bolus of xylazine (0.5 mg/kg dose) or detomidine (0.01 mg/kg dose). The scrotal and inguinal regions were assessed and palpated to verify the presence of a descended testicle [5]. If no testicle could be detected, transrectal examination was performed [3]. After, an ultrasound examination was performed in all cases at the inguinal regions with a portable ultrasound machine, with a multifrequency convex probe 3.5–5 MHz. If the testis was not detected, a transrectal ultrasound examination was also performed with a multifrequency linear probe of 5–7.5 MHz.

### 2.3. Anaesthesia, Analgesia and Surgical Approach

Once the retained testis was localised, an appropriate surgical treatment was planned. The day before surgery, food was withheld for 12–24 h. Peri-operative antibiotic and anti-inflammatory treatments were administered, and the drug type was recorded. According to testis localisation, the following surgical approach was performed: standing flank laparoscopic, ventral midline laparoscopic or open inguinal (or parainguinal) cryptorchidectomy. According to the surgical approach, the patient was prepared for standing or dorsally recumbent anaesthesia.

#### 2.3.1. Standing Flank Laparoscopic Cryptorchidectomy

For a standing laparoscopic procedure, patients were sedated with a bolus of xylazine 0.5 mg/kg or detomidine 0.01 mg/kg and butorphanol tartrate, 0.01–0.025 mg/kg, administered intravenously (IV). After that, a continuous rate infusion (CRI) of xylazine (0.5 µg/kg/min) was administered throughout the procedure [10].

All patients, after achieving an adequate level of sedation, were administered a locoregional anaesthetic aimed at desensitising the lateral abdominal wall to manage intra-operative analgesia during trocar insertion. The locoregional techniques employed were subcutaneous local injection of lidocaine or Transversus Abdominis Plane (TAP) block. The Transversus Abdominis Plane (TAP) block is an interfascial block performed under ultrasound guidance [11]. Following trichotomy and antiseptic preparation of the skin between the last intercostal space and the iliac crest, 0.2 mg/kg of lidocaine was injected subcutaneously approximately 10 cm ventral to the vertebral column and about 5 cm caudal to the last rib. This area corresponded to the first injection site, and the subcutaneous administration of lidocaine facilitated needle insertion for the block. An additional 0.2 mg/kg of lidocaine was injected approximately 10 cm ventral to the vertebral column and 5 cm cranial to the iliac crest (second injection site). At this stage, the ultrasound probe was positioned caudal to the last rib and transverse to the spine, with the marker oriented dorsally. At this location, the external and internal abdominal oblique muscles as well as the transversus abdominis muscle were identified. An echogenic needle (Visioplex, Vygon Italia srl) was inserted in-plane in a dorsoventral direction and advanced toward the fascial plane. After confirming negative aspiration to rule out intravascular placement, a small test bolus of approximately 2 mL of anaesthetic was injected to verify correct hydrodissection and accurate needle positioning. For the second injection site, the probe was positioned cranial to the iliac crest and transverse to the vertebral column, again with the marker oriented dorsally. The needle was inserted using an in-plane technique and advanced in a dorsolateral to ventromedial direction. The injection was performed using the same protocol as for the first site. The total volume administered at each injection site was 0.1 mL/kg of lidocaine (Lidocaine 2%, B. Braun). Moreover, epidural anaesthesia (injection of morphine or metadone with or without lidocaine) was chosen occasionally to provide intra-operative analgesia. Epidural anaesthesia was administered in the first intercoccygeal space using a 22-gauge, 9 cm spinal needle, and the correct placement in the epidural space was confirmed using the hanging drop technique and the absence of resistance during injection [10,12].

For laparoscopy surgical treatment, the flank region was clipped and aseptically prepared, and then the patient was surgically draped. An anatomic site for the first laparoscopic portal was identified at the level of the paralumbar fossa, dorsal to the internal abdominal oblique muscle, between the last rib and the tuber coxae [13,14]. Then, a 10 mm skin incision was made, and a blunt trocar and cannula were introduced into the skin incision, through the abdominal wall, into the peritoneal cavity. A pneumoperitoneum was created with CO_2_ to reach an intrabdominal pressure of 10 to 15 mmHg. The blunt trocar was removed to be replaced with a rigid telescope (0° angle vision, 10 mm diameter) connected to a video camera, and the correct abdominal placement of the first cannula was confirmed.

The second laparoscopic portal was made 4 cm dorsal to the insufflation portal, and a second 10 mm cannula was inserted into the peritoneal space, as previously described [13]. The third portal was created 4 cm ventrally to the first, and another cannula was introduced to serve as a second instrument portal. Once the testis was identified, a 10 mm pair of laparoscopic grasping forceps was introduced through the ventral distal instrument portal to reach and grasp the testis; then, a local injection of lidocaine (i.e., intraparenchymal injection or mesorchium injection) or lidocaine splash was performed. The local injection of lidocaine consisted of mesorchium infiltration with 10 mL of 2% lidocaine through a laparoscopic needle, while the testis was held [10]. Then, the testis was held in place to allow the sealing of the testicular vessels, mesorchium, and vas deferens. A cutting and vessel-sealing device was introduced through the second portal to seal the testicular vessels, mesorchium, and vas deferens until the testis was completely freed, as previously described [13]. Finally, the most ventral portal was enlarged, and the excised testicle was withdrawn using the same grasping forceps. At the end of surgery, the abdomen was deflated, and for all the portals, the muscular incision was closed with one or two cruciate pattern mattress sutures with a 2 USP multifilament absorbable suture, and the skin incision was closed with simple interrupted sutures with 2-0 USP monofilament non-absorbable sutures. In the case of a contralateral descended testis, the latest was removed using a standing open scrotal approach with emasculation of the spermatic cord.

#### 2.3.2. Ventral Midline Laparoscopic Cryptorchidectomy

For a dorsally recumbent procedure, the patients were premedicated with xylazine at a dose of 1.2 mg/kg IV, induced with ketamine at 2.5 mg/kg and midazolam at 0.06 mg/kg IV, and maintained under isoflurane anaesthesia with 100% oxygen [12].

In case a recumbent laparoscopy approach was chosen, the equid was under general anaesthesia and placed in dorsal recumbency, and the ventral abdominal area was clipped, aseptically scrubbed and draped for the surgical procedure [15]. A skin incision was made at the umbilicus, and the linea alba was penetrated. A teat cannula was placed into the peritoneum, an insufflation tube was connected, and the abdomen was distended with CO_2_. Then, a laparoscopic cannula was inserted, and the laparoscope (10 mm diameter, 0°) was connected. Then, the surgical table was tilted, placing the head of the horse down into a 25° Trendelenburg position. The laparoscope was directed caudally, and the inguinal area was inspected. The instrument portals were placed 10 cm cranial to the external inguinal ring and 10 cm lateral to the midline, and the accessory trocar-cannula was placed under direct laparoscopic guidance [15]. Once the testis was detected, it was grasped with claw forceps, and a local injection of lidocaine (i.e., intraparenchymal injection or mesorchium injection) or lidocaine splash was performed. Then, a vessel-sealing device was placed through the ipsilateral portal for haemostasis. The testis was removed through the enlarged portal. Then, the abdomen was decompressed, and the surgical table was returned to horizontal. The muscular layer of the laparoscopic portals was closed with a cruciate interrupted pattern, a 2 USP multifilament absorbable suture. Subcutaneous tissue was closed with 2-0 USP monofilament absorbable sutures in a simple continuous pattern. The skin incisions were apposed with a simple interrupted suture pattern with 2-0 monofilament non-absorbable sutures.

#### 2.3.3. Open Inguinal Cryptorchidectomy

In case of an inguinal retained testis, an open inguinal cryptorchidectomy under general anaesthesia was performed in dorsal recumbency. For a dorsally recumbent procedure, the patients were premedicated with xylazine at a dose of 1.2 mg/kg IV, induced with ketamine at 2.5 mg/kg and midazolam at 0.06 mg/kg IV, and maintained under isoflurane anaesthesia with 100% oxygen [12]. As described in the literature [16], a skin incision was made over the inguinal area, at the side of the retention, and then the superficial inguinal ring was reached. The vaginal process was identified, everted, and opened to locate and remove the testis. The superficial inguinal ring was closed with cruciate-pattern sutures with a 2 USP multifilament absorbable suture, the subcutis with a simple continuous pattern suture with 2-0 USP monofilament absorbable suture, and the skin incision with simple interrupted sutures with 2-0 USP monofilament non-absorbable sutures [16].

For each procedure, intra-operative complications were recorded.

### 2.4. Statistical Analysis

Descriptive statistical analyses were performed, and data were expressed as frequency and percentage.

## 3. Results

A total of 37 clinical records were included. Horses were mainly represented (36/37, 97%), while only one was a donkey (1/34, 3%). The majority were males (36/37, 97%), while one was intersex (1/37, 3%) who was a pseudohermaphrodite. The median age was 3 years (range, 2–20 years). The Quarter Horse-type breeds were mostly represented (Quarter Horse 14/37, 38%; Appaloosa 1/37, 3%). The remaining equids were of less represented breeds: Italian Warmblood, 5/37 (14%); Arabian, 1/37 (3%); Italian Drafthorse, 1/37 (3%); Hannover, 1/37 (3%); Friesian, 1/37 (3%); Thoroughbred, 1/37 (3%); Anglo-Arabian, 1/37 (3%); Maremmano, 1/37 (3%); Standardbred, 1/37 (3%); French Warmblood, 1/37 (3%); Holland Warmblood, 1/37 (3%). Six out of thirty-seven (16%) horses were of unknown breed.

**Figure 1:**
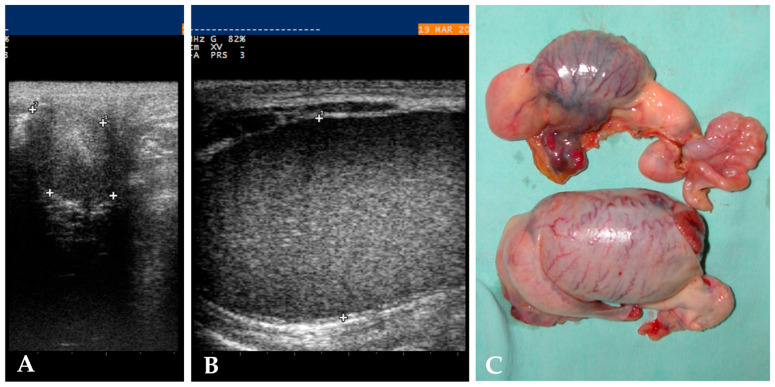
Ultrasonographic aspect of the left intra-abdominal testis ((**A**), 25.8 × 34.7 mm) (transrectal ultrasound), of the right scrotal testis ((**B**), 36.8 × 58.7 mm) (inguinal ultrasound), and of the removed gonads (**C**) of a Quarter Horse stallion.

Historical data were available for 13/37 (35%). Four out of thirteen (31%) were unilaterally castrated cryptorchid horses, and three out of thirteen (23%) were sons of the same stallion. Diagnostic examination data were missing for 6/37 (16%) records. For the horses that had ultrasound examinations performed, 27/31 (81%) had the location of the testicle identified (Figure 1). In 3/31 (10%) cases, the testis was visualised only after a direct laparoscopic procedure, and in 1 case (1/31, 3%) through the inguinal palpation.

Unilateral cryptorchidism was identified in 28/37 (76%), and bilateral cryptorchidism was identified in 8/37 (22%), while in 1 case (1/37, 3%), monorchidism was diagnosed. Monorchid testis showed associated spermatic structures (i.e., deferent duct and gubernaculum testis) and the absence of testicular tissue on histopathology, located in the inguinal region. The right testis (16/28; 57%) was more commonly retained compared to the left (11/28; 39%), and no data was recorded for one case (1/37; 4%). Testis locations were inguinal in 21/37 (57%) and abdominal in 16/37 (43%).

Surgical approaches were recorded for 36/37 (97%). Cryptorchidectomy was primarily performed using a laparoscopic surgical technique (23/36, 64%), while the inguinal open approach was performed in 13/36 (36%) of the cases. For the laparoscopic approach, the standing flank technique was performed in the majority of cases (19/23 (83%)), while the dorsally recumbent laparoscopy technique was performed in 4/23 (17%). The dorsally recumbent laparoscopy technique was preferred for uncooperative horses and in young and small-sized equid.

The anaesthesia plan (Table 1) was recorded for the majority of the records (33/37, 89%), and 19/33 (58%) patients received a local anaesthesia in addition to the anaesthesia plan. Most of the local anaesthesia (7/19, 37%) was administered with a local injection of lidocaine only (i.e., intraparenchymal injection or mesorchium injection); in 5/19 (26%) patients, local injection of lidocaine and epidural anaesthesia was used (with the injection of morphine or metadone with or without lidocaine); in 4/19 (21%) patients, lidocaine splash and local injection of lidocaine was used; and in 2/19 (11%) patients, only epidural anaesthesia was used. In only one patient, a Transversus Abdominis Plane (TAP) block was performed (1/19, 5%).

As pre-operative medical treatments, the combination of procaine-benzylpenicillin and flunixin meglumine was commonly administered (16/37, 43%), and, less frequently, the following treatments were administered: the combination of procaine-benzylpenicillin, flunixin meglumine and gentamicin (9/37, 24%); the combination of procaine-benzylpenicillin and phenylbutazone (2/37, 5%); procaine-benzylpenicillin only (2/37, 5%); gentamicin and flunixin meglumine (1/37, 3%); ceftiofur and flunixin meglumine (1/37, 3%); flunixin meglumine only (1/37, 3%); and the combination of ampicillin, gentamicin and flunixin meglumine (1/37, 3%). For 4/37 (11%) records, pre-operative treatment data were missing.

As post-operative treatment, the combination of procaine-benzylpenicillin (22,000 UI/kg, IM) and flunixin meglumine (1.1 mg/kg, IV) was commonly administered (16/37, 43%), and less frequently, the following treatments were administered: flunixin meglumine only (10/37, 27%); the combination of procaine-benzylpenicillin and phenylbutazone (3/37, 8%); the combination of gentamicin and flunixin (2/37, 5%); procaine-benzylpenicillin, flunixin meglumine and gentamicin (2/37, 5%); ceftiofur, gentamicin and phenylbutazone (1/37, 3%); ceftiofur and flunixin meglumine (1/37, 3%); and ampicillin, gentamicin and flunixin meglumine (1/37, 3%). For 1/37 (3%) records, pre-operative treatment data were missing.

No intra-operative complications were recorded in 34/37 cases (92%). In 3/37 (8%) cases, the intra-operative complications were the following: challenging identification and testis removal (1/3, 33%), incorrect laparoscopic portals (1/3, 33%), and surgical plan shift from a standing laparoscopic approach to open inguinal orchiectomy (1/3, 33%).

In the outcome, all cases were discharged, and the median hospitalisation was 2 days (range, 1–8).

## 4. Discussion

The findings reported in the present study will advance our understanding of this widely prevalent condition in equids and aid in selecting the most suitable clinical, diagnostic and surgical strategies. This study aimed to validate previous results and expand knowledge by investigating a distinct population of equids referred to specialised centres, thereby enhancing the applicability and robustness of both current and past findings. This research places particular focus on clinical protocols for determining the optimal diagnostic method for localising retained testes, as well as on the surgical techniques and local anaesthesia approaches employed during treatment. In our study, cryptorchidism was commonly seen in Quarter Horses, with evidence suggesting a possible genetic contribution. Ultrasound was a valuable tool for locating undescended testicles in both the abdominal and inguinal areas. Anaesthesia typically involved local blocks, primarily through infiltration of the testicular parenchyma and mesorchium. Flank laparoscopy was the preferred surgical technique due to its advantages over dorsally recumbent laparoscopy and open inguinal orchiectomy. Positive outcomes with few short-term complications align with literature reports and help improve clinical, diagnostic, surgical and anaesthesia management of cryptorchidism in equids.

Western riding horses (namely, Quarter Horse and Appaloosa) were overrepresented among others, according to the literature, highlighting the highest incidence for these breeds [1,2]. Interestingly, three horses were sons of the same Quarter Horse stallion, which suggests a genetic component in cryptorchidism aetiology, as previously reported [3]. In our population, a case of pseudohermaphroditism was reported: the horse had masculinised external genitalia and retained testes but was XX at the karyotype. Male pseudohermaphroditism is the most common sexual disorder found in horses, usually associated with intra-abdominal or inguinal testes, ambiguous external genitalia and stallion-like behaviour [1].

In our population, the prevalence of hemicastration was 31%, slightly lower than the previously reported value (41%) [1,17]. In horses with no clear castration history, it can be clinically challenging to distinguish between bilateral or hemicastrated cryptorchid horses and geldings with stallion-like behaviour. Endocrine testing is helpful to differentiate cryptorchid horses and the presence of gonadal tissues, with the quantification of hormones like testosterone or Anti-Müllerian Hormone (AMH). AMH is produced by Sertoli cells and represents an excellent biomarker to detect the presence of testicular tissue, and in cryptorchid stallions, serum AMH is higher than in stallions or geldings [1]. In our population, cryptorchidism was diagnosed primarily by ultrasound (87%), and for any case, hormone analysis was performed. Ultrasound diagnosis findings were in line with a previous study that highlighted how the systematic use of ultrasound, in both the abdominal and inguinal regions, successfully confirmed cryptorchidism, initially diagnosed by palpation, in up to 97.5% of cases [4,18]. Consistent with the previous literature, unilateral right cryptorchidism, located in the inguinal canal, was mainly reported [1,5]. Our findings show a case of monorchidism, diagnosed on laparoscopy and confirmed by histopathology. There were a limited number of previously published case reports and small case series that diagnosed monorchidism after exploratory surgery [6,19]. According to our findings, it was reported that monorchidism was likely to be associated with spermatic structures and the absence of testis tissue [6]. Conversely, Sinovich and colleagues (2025) [19] recently reported a clinical case with a diagnosis of monorchidism: the testicle was retained intra-abdominally, and histopathology was consistent with testicular tissue displaying a Sertoli cell-only pattern with bipolar ductal structures. The exact causes of monorchidism are still unclear; however, molecular and cytogenetic analysis reported an elevation of the rate of micronuclei, as evidence for genome instability, which might have been involved in the failure of normal testicular development and descent [6,19].

Standing flank laparoscopy was preferred by surgeons in this study. This technique offers advantages over a recumbent laparoscopy and inguinal open cryptorchidectomy, as previously reported [2]. Cryptorchidectomy was mainly achieved with a laparoscopy surgical technique, with a standing flank approach performed in the majority of cases (83%), compared to dorsally recumbent approaches. Nowadays, standing laparoscopic cryptorchidectomy has been the most reported technique because it provides excellent visualisation of the intra-abdominal pathway undertaken by the descended testis, which then avoids general anaesthesia [14,15,16]. In a small portion of the population study, dorsal recumbency was preferred over the standing approach, mainly related to horse behaviour and equid size. As previously reported, a standing approach may not be ideal for uncooperative horses, where patient movement may not facilitate the procedure or even the safe removal of the descended testis [14,15,16]. Moreover, in young colts or small ponies and donkeys, size limitations may preclude a standing approach [14,15,16]. A study found that laparoscopic cryptorchidectomy was associated with disadvantages compared with conventional open cryptorchidectomy [20]. This result was mainly related to the longer surgical preparation time for the laparoscopic setting compared to the traditional one. Laparoscopic surgery is also technically more challenging, and total surgery time may be strongly influenced by the surgeon’s experience and skills [20].

Local lidocaine injection into the retained genitalia remnants was the most commonly chosen local anaesthesia technique. This approach is rapid, simple to perform, and associated with a low complication rate, which likely explains its preference [10]. In one case, the TAP block—a regional anaesthesia technique commonly used in abdominal surgery for humans and small animals—was performed [11,19,21]. Recently adopted in equine anaesthesia, it requires ultrasound guidance and skilled operators [21,22]. The TAP block effectively manages perioperative pain, reduces systemic drug use and serves as rescue analgesia post-operatively [21,22,23].

Finally, all cases reported had a favourable outcome, a low rate of intra-operative complications and a short hospitalisation period; however, no data were available for short-term complications in our records. This was consistent with the current literature, which reported mainly short-term complications such as fever and swelling of the surgical site [2,7,24,25,26,27,28]. The present findings highlight complications mainly related to the surgical procedure, such as challenging testis removal and incorrect surgical laparoscopic technique [29].

Previous studies found challenging identification and grasping of the structures associated with the testis (gubernaculum testis, epididymis) as the main intra-operative complications in horses with inguinal cryptorchidism, treated with open inguinal orchiectomy [5,25,26,27,28]. Contrary to our findings, most of the studies reported moderate self-limiting bleeding from the spermatic cord, mild fever, wound emphysema and oedema [3,5,26,27,28].

Pre- and post-operative treatments were primarily represented by the combination of a broad-spectrum antibiotic and an NSAID, consistent with previous reports on the prophylactic use of antimicrobials to avoid secondary infections in elective surgery [1,20].

The present research shows some limitations due to the retrospective nature of the study. The lack of some of the history and segmental information could have negatively influenced our results, probably through inaccurate clinical reports and challenging interactions with the owners [30]; at the same time, this highlights the need to collect accurate anamnestic data, which is essential for a prompt and appropriate diagnosis and treatment approach. Moreover, a limitation of the present study is the lack of information on surgical and anaesthesia time, which would have added precious information to critically analyse and compare outcomes and complications [20].

## 5. Conclusions

In our retrospective study, cryptorchidism is a common pathology among Quarter Horse types. In particular, three horses were sons of the same quarter horse stallion, suggesting a genetic component in aetiology. Ultrasonography proved to be a valuable tool for localising retained testes, confirming its utility in both abdominal and inguinal locations. An anaesthesia plan commonly includes a local anaesthesia block, although in the majority of cases, it involves a local injection for infiltration of the testicular parenchyma and mesorchium. Standing flank laparoscopy was mostly performed and preferred in our study over recumbent laparoscopy and inguinal open orchiectomy. The favourable outcome was consistent with the literature, reporting a low rate of short-term complications. The findings would improve our knowledge of this condition, which is widely spread among equids and is useful for choosing the most appropriate clinical, diagnostic and surgical approaches.

## Figures and Tables

**Table 1:** Local anaesthesia technique data expressed as prevalence and percentage.

Local Anaesthesia Technique 19/33 (58%)	Prevalence and Percentage
Local injection of lidocaine only	7/19, 37%
Local injection and epidural anaesthesia	5/19, 26%
Local injection and splash	4/19, 21%
Epidural anaesthesia only	2/19, 11%
TAP block	1/19, 5%

## Data Availability

The original contributions presented in this study are included in the article. Further inquiries can be directed to the corresponding author.
